# Book Notices

**Published:** 1897-12

**Authors:** 


The Living Age for 1898.—In another column will be found a prospectus of this standard periodical. Founded by Eliakim Littell in 1844, it has steadily maintained the reputation gained with its earliest issues of being the most complete representative of foreign thought as expressed by its greatest exponents. It is today a faithful reflection of almost all that is substantial and truly valuable in the passing literature of the world, embracing as it now does in its Monthly Supplement, American as well as foreign literature. While its pages show the same wise and judicious discrimination which has ever characterized its editorial management, the scope of the magazine has been widened, its size increased and its price reduced, so that increasing years seems only to add to its vigor and value. To those whose means are limited it must meet with especial favor, for it offers them what could not otherwise be obtained except by a large outlay. Intelligent readers who want to save time and money will find it invaluable. *The Living Age* is published weekly, and the price is now but $6.00 a year. To all new subscribers for 1898 are offered free the eight numbers of 1897, containing the opening chapters of the new serial, "With All Her Heart," described in the prospectus.

---

Among the topics discussed by the editor of the *American Monthly Review of Reviews* in the November number of that periodical are the Greater New York political campaign, other municipal elections, the "Referendum" in American elections, the foreclosure of the Union Pacific, the crisis in Spain, recent events in Cuba, England's attitude towards bimetallism, the proposed international sealing conferences, politics in Eastern Europe, Australian federation, and the careers of Charles A. Dana, George M. Pullman and Neal Dow.

FAMOUS CHRISTMAS STORY TELLERS.—The Christmas *Ladies' Home Journal* will have Christmas stories by Mary E. Wilkins, Ruth McEnery Stuart, Hamlin Garland, Mrs. A. D. T. Whitney, Mrs. Mark Morrison and Lilian Bell.

